# 新型Ⅰ类和Ⅱb类选择性HDAC抑制剂甲磺酸普依司他治疗弥漫大B细胞淋巴瘤的体外药效学和作用机制研究

**DOI:** 10.3760/cma.j.issn.0253-2727.2022.09.007

**Published:** 2022-09

**Authors:** 婕 王, 艾琳 赵, 赫 李, 林玉 杨, 俐娟 陈, 挺 牛

**Affiliations:** 1 四川大学华西医院血液内科，成都 610041 Department of Hematology, West China Hospital, Sichuan University, Chengdu 610041, China; 2 四川大学生物治疗国家重点实验室，成都 610041 State Key Laboratory of Biotherapy and Cancer Center, West China Hospital, Sichuan University, Chengdu 610041, China

**Keywords:** 组蛋白脱乙酰基酶抑制剂, 淋巴瘤，大B细胞，弥漫性, 体外药效学, 作用机制, Histone deacetylase inhibitors, Lymphoma, large B-cell, diffuse, In vitro pharmacodynamic, Mechanism

## Abstract

**目的:**

探究新型Ⅰ类和Ⅱb类选择性组蛋白去乙酰化酶（HDAC）抑制剂甲磺酸普依司他（PM）对弥漫大B细胞淋巴瘤的体外抑制活性及作用机制。

**方法:**

MTT法检测PM对细胞增殖的影响，流式细胞术检测PM对细胞周期、细胞凋亡的影响，Western blot法检测HDAC底物乙酰化水平、细胞周期蛋白、凋亡相关蛋白和癌基因蛋白的表达。

**结果:**

PM对淋巴瘤细胞株SUDHL-4和SUDHL-6的增殖具有明显抑制作用，能够上调HDAC底物H3、H4和α-tubulin乙酰化水平。在细胞周期实验中，PM可诱导SUDHL-4、SUDHL-6细胞G_0_/G_1_期阻滞，Western blot显示PM能显著下调细胞周期蛋白依赖性激酶Cdk2、Cdk4、Cdk6及细胞周期蛋白Cyclin D1、Cyclin E的表达，上调CDK抑制蛋白p21的表达。在细胞凋亡实验中，PM可诱导SUDHL-4和SUDHL-6细胞凋亡，Western blot显示PM通过激活Caspase3激酶和影响抗凋亡蛋白Bcl-2促进细胞内源性凋亡。此外，PM也能够下调癌基因标志蛋白MYC、IKZF1、IKZF3的表达。

**结论:**

PM在体外对弥漫大B细胞淋巴瘤包括双打击淋巴瘤具有高效的生物活性，为PM应用于临床治疗提供了有价值的实验依据。

## Abstract (en)

**Objective:**

To investigate the in vitro inhibitory activity of a novel class Ⅰ and Ⅱb selective histone deacetylase（HDAC）inhibitor, purinostat mesylate（PM）, in diffuse large B-cell lymphoma and its mechanism.

**Methods:**

The 3-（4,5-dimethylthiazol-2-yl）-2,5-diphenyl tetrazolium bromide method was used to detect the effect of PM on cell proliferation. The effects of PM on cell cycle and apoptosis were detected by flow cytometry. The acetylation levels of HDAC substrate, cell cycle protein, apoptosis-related protein, and oncogene protein expression were detected by Western blot.

**Results:**

PM significantly inhibited the proliferation of lymphoma SUDHL-4 and SUDHL-6 cells and increased the acetylation levels of HDAC substrates H3, H4, and α-tubulin. In cell cycle experiments, PM induced G_0_/G_1_ phase arrest in SUDHL-4 and SUDHL-6 cells. Western blot experiment showed that PM could significantly downregulate the expression of cyclin-dependent kinases Cdk2, Cdk4, Cdk6, cyclin D1, and cyclin E and upregulate the expression of CDK inhibitor protein p21. In the apoptosis experiment, PM could induce the apoptosis of SUDHL-4 and SUDHL-6 cells. Western blot experiment demonstrated that PM promoted endogenous apoptosis by activating caspase-3 kinase and affecting antiapoptotic protein Bcl-2. In addition, PM could downregulate the expression of oncogene marker proteins MYC, IKZF1, and IKZF3.

**Conclusion:**

PM has an efficient biological activity in vitro for diffuse large B-cell lymphoma, including double-hit lymphoma, and provides valuable experimental evidence for PM in clinical treatment.

弥漫大B细胞淋巴瘤（DLBCL）是最常见的淋巴瘤亚型，我国每年约3万新发病例，占所有淋巴瘤的30％～40％[1]–[2]。在利妥昔单抗时代，一线免疫化疗后仍有40％～50％的患者治疗后出现疾病难治或复发[3]，复发或难治患者预后较差，通过挽救性化疗或自体造血干细胞移植治疗，仅10％的患者能最终治愈[4]–[9]。其中部分病例涉及MYC、BCL2和（或）BCL6基因重排，被称为双打击淋巴瘤（DHL）或三打击淋巴瘤（THL）。DHL在DLBCL中所占比例虽不超过12％[10]，但侵袭性强，常规化疗预后极差，目前尚无标准治疗方案。因此，DLBCL包括DHL在治疗方面仍存在未满足的需求，迫切需要开发新型靶向药物和创新性疗法。

组蛋白去乙酰化酶（histone deacetylase，HDAC）是表观遗传学领域用于肿瘤临床研究最成功的一类靶点，共分为Ⅰ、Ⅱ、Ⅲ、Ⅳ四类，研究表明，在血液系统肿瘤中常发生HDAC Ⅰ类和Ⅱ类酶的过表达或失调[11]。靶向抑制HDAC的活性可抑制肿瘤细胞增殖分化、抑制肿瘤迁移或促进肿瘤细胞凋亡等[12]。目前全球已有5个HDAC抑制剂获批上市[13]–[14]，其中4个适应证均为复发难治性外周T细胞淋巴瘤，1个获批用于复发难治性多发性骨髓瘤的联合治疗，多数为非选择型的HDAC泛抑制剂，常见的脱靶效应导致药物不良反应较多。新型Ⅰ类和Ⅱb类选择性HDAC抑制剂甲磺酸普依司他（purinostat mesylate，PM）2018年11月获国家药品监督管理局批准，目前正在四川大学华西医院开展Ⅰ期临床试验（CXHL1800174），获批的适应证为复发或难治的以B细胞相关肿瘤为主的血液系统肿瘤。为探讨PM在DLBCL包括DHL中的潜在临床应用价值，我们进行了如下初步研究。

## 材料与方法

1. 主要试剂：PM由四川大学生物治疗国家重点实验室提供，批号PMF20170901；纯度：98.67％；含量95.80％。帕比司他（LBH589）购自大连美仑公司。一抗Ac-α-tublin购自美国Santa Cruz Biotechnology 公司，Bcl-2购自美国Sigma-Aldrich公司，c-Myc、IKZF1购自英国Abcam公司，Ac-H3、Ac-H4、Cleaved caspase3、PARP-1、IKZF3购自成都正能生物技术有限责任公司，p21、Cdk2、Cdk4、Cdk6、CyclinD1、CyclinE购自美国Cell Signaling Technology公司。GAPDH、鼠抗人β-actin单克隆抗体，HRP标记羊抗兔和羊抗鼠二抗购自北京中杉金桥生物技术有限公司。膜联蛋白V（Annexin V）/碘化丙锭（PI）双染试剂盒、PI及RNase均购自大连美仑公司。

2. 细胞株：人DLBCL细胞株SUDHL-4、SUDHL-6（DHL亚型）来自美国典型培养物保藏中心（American Type Culture Collection，ATCC），由四川大学生物治疗国家重点实验室细胞库培养保种。细胞培养于含10％胎牛血清、100 U/ml青霉素和100 mg/L链霉素的RPMI 1640培养液中，在37 °C、5％CO_2_、饱和湿度的恒温培养箱中培养，定期传代，取对数生长期细胞用于实验。

3. MTT法检测药物对细胞增殖的影响：选用对数生长期的肿瘤细胞，将细胞按（1～2）×10^4^个/100 µl接种于96孔板中，按浓度梯度加入PM或LBH589（1 000、200、40、8、1.6、0.32、0.064 nmol/L），对照孔加入等体积稀释相同比例的DMSO溶液，置于37 °C、5％CO_2_、饱和湿度培养箱中培养至72 h，每孔加入20 µl的MTT溶液，在培养箱孵育4 h后每孔加入MTT溶解液100 µl，次日用酶标仪测定570 nm处的吸光值，采用Graphpad prism 5.0软件计算细胞增殖抑制率，拟合半抑制浓度（IC_50_）值。

4. 细胞周期的流式细胞术检测：收集不同浓度PM（0.25、0.5、1 nmol/L）处理24 h后的细胞悬液，500×*g*离心3 min，PBS洗涤细胞2次，70％冰乙醇固定，加PI染液避光染色约10 min后进行流式细胞术检测，流式细胞术结果采用ModFit软件进行细胞群体各周期比例分析。

5. 细胞凋亡的流式检测：收集不同浓度药物处理48 h后的细胞悬液，500×*g*离心3 min，PBS洗涤细胞2次。加结合缓冲液（binding buffer）重悬细胞后，每个流式管中加入5 µl体积的Annexin Ⅴ染液，室温下避光染色10 min，再加入5 µl的PI染料染色，避光放置，1 h内上流式细胞仪检测。

6. Western blot分析：分别收集药物处理组及未处理组细胞，加入细胞裂解液混匀后冰上裂解30 min，离心收集上清，BCA法检测蛋白浓度，然后进行SDS-聚丙烯酰胺凝胶电泳后转膜至PVDF膜，50 g/L脱脂牛奶室温封闭2 h，一抗4 °C孵育过夜，次日洗涤后加入相应二抗共孵育1 h，洗涤后进行X光胶片显影，采用Image J分析软件进行灰度扫描。

7. 统计学处理：采用Graphpad Prism 6.0软件进行数据分析，多组数据间比较采用单因素方差分析（ANOVA），采用tukey的多组比对分析法进一步分析特定两组之间差异的显著性。两组数据间比较采用*t*检验，*P*<0.05为差异有统计学意义，实验数据以平均值±标准差表示。

## 结果

1. PM在体外对SUDHL-4和SUDHL-6细胞增殖的影响：PM对DLBCL细胞株SUDHL-4和SUDHL-6（DHL亚型）具有明显抑制作用，与同类药物LBH589相比，其IC_50_值分别为（0.333±0.279）nmol/L对（3.431±0.588）nmol/L和（0.557±0.195）nmol/L对（4.005±0.372）nmol/L（实验重复3次）。进一步对SUDHL-4、SUDHL-6进行化合物的细胞毒活性分析，测定药物作用72 h后发挥细胞毒活性的半数致死量（LD_50_）值，结果显示，与LBH589相比，PM对SUDHL-4、SUDHL-6细胞株具有明显细胞毒性，其LD_50_值分别为（2.733±0.561）nmol/L对（17.140±0.580）nmol/L和（1.278±0.198）nmol/L对（9.718±0.540）nmol/L（实验重复3次）。

2. PM对组蛋白去乙酰化酶的抑制作用：组蛋白H3、H4作为HDAC Ⅰ类酶的底物，其乙酰化状态（Ac-H3、Ac-H4）可用来衡量HDAC Ⅰ类酶活性；微管蛋白α-tubulin是HDAC6的特异性底物，其乙酰化的状态（Ac-α-tubulin）常用来衡量HDAC Ⅱb类酶活性。在SUDHL-4和SUDHL-6细胞中加入设定浓度的PM处理6 h后收集细胞提取蛋白，Western blot法检测标志性蛋白Ac-H3、Ac-H4和Ac-α-tubulin的表达情况。结果显示，对于SUDHL-4细胞，0.3～1 nmol/L浓度的PM就可明显诱导组蛋白H3或H4乙酰化水平升高，30～100 nmol/L浓度可明显诱导α-tubulin乙酰化水平升高（图1）。在SUDHL-6细胞中也得到类似的结果，0.3～1 nmol/L浓度的PM可明显诱导组蛋白H3或H4乙酰化水平升高，3～10 nmol/L浓度可明显诱导α-tubulin乙酰化水平升高（图2）。细胞实验结果显示了PM对HDAC Ⅰ类和Ⅱb类的高抑制活性，与同类上市药物LBH589比较，PM对HDAC Ⅰ类的抑制活性明显优于LBH589。

**图1:**
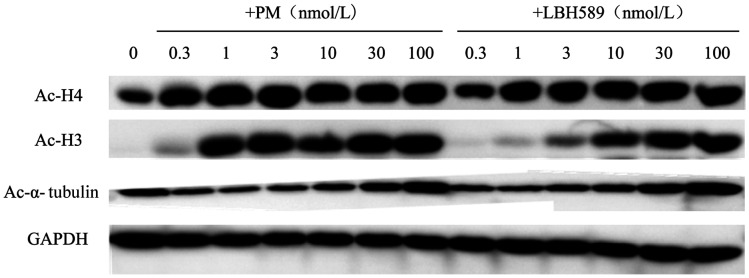
PM作用于SUDHL-4细胞6 h后对组蛋白H3、H4和α-tubulin的乙酰化水平的影响 PM：甲磺酸普依司他；LBH589：帕比司他

**图2:**
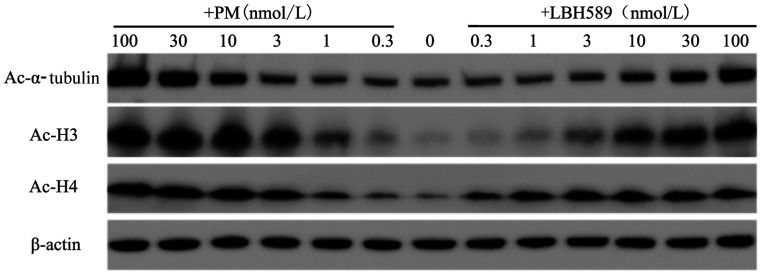
PM作用于SUDHL-6细胞6 h后对组蛋白H3、H4和α-tubulin的乙酰化水平的影响 PM：甲磺酸普依司他；LBH589：帕比司他

3. PM对淋巴瘤细胞G_0_/G_1_期及细胞周期相关蛋白的影响：不同浓度（0.25、0.5、1 nmol/L）的PM处理SUDHL-4、SUDHL-6细胞24 h，70％乙醇固定后行PI染色，流式细胞仪进行细胞周期分析。结果显示，PM可诱导SUDHL-4、SUDHL-6细胞G_0_/G_1_期阻滞，且呈剂量依赖性（图3）。0.5 nmol/L的PM能显著诱导SUDHL-4和SUDHL-6细胞G_0_/G_1_期阻滞，G_0_/G_1_期细胞所占比例分别为64.89％、74.04％，而溶剂对照组G_0_/G_1_期细胞所占比例分别为46.92％、54.81％。

**图3:**
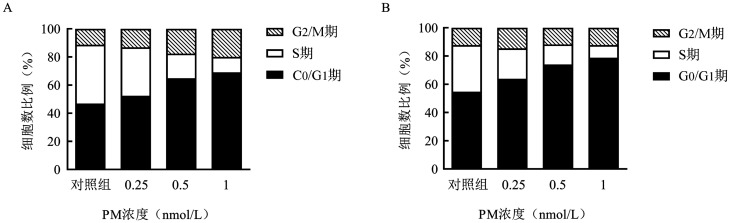
PM诱导SUDHL-4（A）、SUDHL-6（B）细胞G_0_/G_1_期阻滞情况 PM：甲磺酸普依司他

通过Western blot研究G_0_/G_1_期相关细胞周期蛋白及细胞周期蛋白依赖性激酶（CDKs）抑制剂p21的表达。结果显示，PM能显著下调细胞周期蛋白依赖性激酶Cdk2、Cdk4、Cdk6及细胞周期蛋白Cyclin D1、Cyclin E的表达，浓度依赖地上调CDK抑制蛋白p21的表达（图4）。

**图4:**
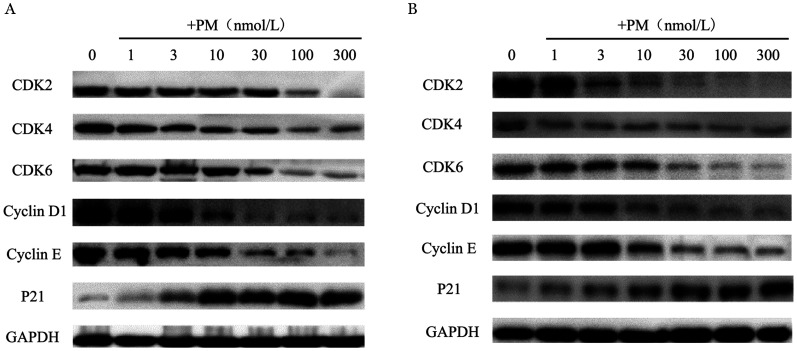
甲磺酸普依司他（PM）对SUDHL-4（A）和SUDHL-6（B）细胞G_0_/G_1_期相关蛋白水平的影响

4. PM对淋巴瘤细胞凋亡及凋亡相关蛋白表达的影响：用不同浓度（0.3、3、30 nmol/L）的PM处理SUDHL-4、SUDHL-6细胞48 h后，流式细胞术分析细胞凋亡情况。结果显示，30 nmol/L的PM能明显引起SUDHL-4、SUDHL-6细胞凋亡，凋亡细胞所占比例分别为93.40％、93.60％；溶剂对照组凋亡细胞所占比例分别为5.73％、2.06％，远低于药物作用组（图5）。

**图5:**
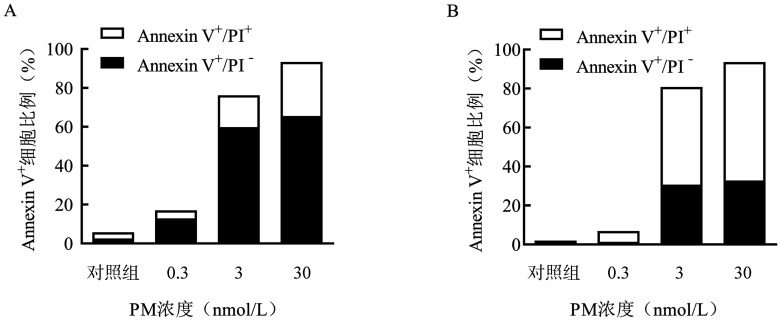
甲磺酸普依司他（PM）诱导SUDHL-4（A）、SUDHL-6（B）细胞凋亡情况

PM按照设定浓度处理SUDHL-4和SUDHL-6细胞48 h，Western blot法检测其凋亡蛋白的表达情况。结果显示，PM处理SUDHL-4和SUDHL-6细胞48 h后，Cleaved-Caspase3和PARP-1表达上调，说明PM激活了Caspase3激酶。此外抗凋亡蛋白Bcl-2表达下调，表明PM能诱导SUDHL-4和SUDHL-6细胞发生内源性凋亡（图6）。

**图6:**
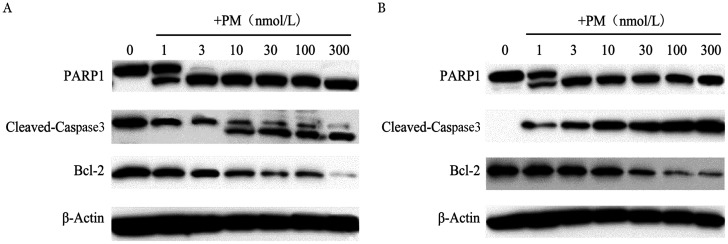
甲磺酸普依司他（PM）对SUDHL-4（A）和SUDHL-6（B）细胞PARP-1、Cleaved-Caspase3、Bcl-2蛋白水平的影响

5. PM对癌基因标志蛋白MYC、IKZF1、IKZF3表达的影响：以设定浓度的PM处理SUDHL-4和SUDHL-6细胞24 h后，收集细胞提取蛋白，Western blot法检测癌基因标志性蛋白MYC、IKZF1和IKZF3的表达情况。结果显示，PM能够浓度依赖性地下调SUDHL-4和SUDHL-6细胞MYC、IKZF1、IKZF3的表达（图7）。

**图7:**
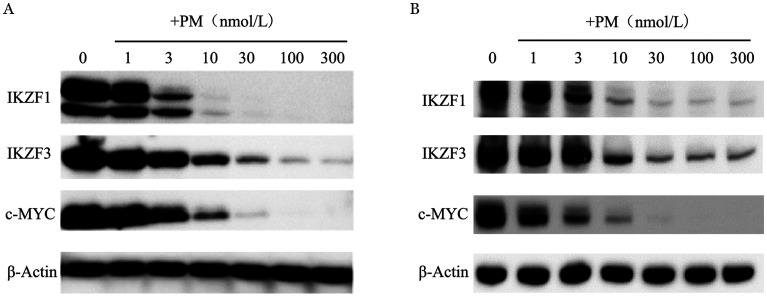
甲磺酸普依司他（PM）作用于SUDHL-4（A）和SUDHL-6（B）细胞24 h后对IKZF-1、IKZF-3和c-MYC的影响

## 讨论

近年来，HDAC在肿瘤中的作用受到广泛关注，HDAC的功能为催化组蛋白及部分非组蛋白赖氨酸残基的去乙酰化，在细胞内的表观遗传和转录后修饰中发挥重要作用[15]–[16]。研究发现，在肿瘤细胞中常发生HDAC高表达，导致表观遗传失控和胞质内重要功能蛋白失调[11]。HDAC抑制剂能够通过恢复组蛋白的乙酰化水平激活抑癌基因，并通过诱导多种胞质蛋白乙酰化抑制肿瘤细胞增殖，促使肿瘤细胞凋亡[12]。研究表明，HDAC中Ⅰ类和Ⅱ类与肿瘤关系密切，Ⅱ类又分为Ⅱa和Ⅱb，其中Ⅱa类和Ⅳ类酶与细胞运动、细胞能量代谢、机体免疫调节等功能密切相关[17]。目前全球已有5个HDAC抑制剂获批上市[13]–[14]，中国仅有1个HDAC抑制剂获批上市，其中3款为HDAC泛抑制剂，因存在对Ⅱa和Ⅳ类酶的抑制而导致药物不良反应较多，LBH589可能引起严重心脏毒性和严重腹泻。设计合成HDAC Ⅰ类和Ⅱb类高选择性抑制剂可以降低HDAC抑制剂治疗肿瘤的不良反应。PM是高选择性Ⅰ类和Ⅱb类HDAC抑制剂，且酶抑制活性优于LBH589，可避免对Ⅱa和Ⅳ亚型抑制引起的毒性。

DLBCL是最常见的B细胞淋巴瘤亚型，我国每年新增淋巴瘤患者约10万人，其中DLBCL约占三分之一[1]–[2]。在利妥昔单抗时代，常规免疫化疗后40％～50％的患者出现难治或复发[3]。复发或难治性患者预后较差，通过传统挽救性化疗或自体造血干细胞移植治疗后，仅约10％患者能最终治愈[4]–[9]。复发或难治性DLBCL常为特殊亚型或存在预后不良因素，其中部分病例涉及由于MYC、BCL2和（或）BCL6基因重排导致的MYC、BCL2和（或）BCL6过表达，这类亚型被2017年WHO淋巴瘤第4版修订版分类正式命名为高级别B细胞淋巴瘤伴MYC、BCL2和（或）BCL6重排[18]，俗称DHL或THL，该类型淋巴瘤中癌基因MYC、BCL和（或）BCL6高度活化，侵袭性高，生存率低，目前尚无标准治疗方案，尽管进行强化联合化疗（RTX+Hyper-CVAD、RTX+DA-EPOCH等）甚至自体造血干细胞移植，中位总生存期仅1～2年[19]–[21]。因此，DLBCL包括DHL在治疗方面仍存在未满足的需求，迫切需要开发新型靶向药物和创新性疗法以提高疾病治愈率，改善疾病治疗现状。

我们的体外实验表明，PM对DLBCL包括DHL细胞系具有很强的抗增殖活性和细胞毒性，并优于同类药物LBH589。PM能够上调DLBCL和DHL细胞系组蛋白H3、H4和α-tubulin的乙酰化水平，诱导发生G_0_/G_1_期周期阻滞从而抑制淋巴瘤细胞增殖，以浓度依赖的方式诱导淋巴瘤细胞凋亡。Western blot显示PM可能通过影响CDK-Cyclin复合物活性诱导细胞G_0_/G_1_期阻滞，并通过激活Caspase3激酶和影响抗凋亡蛋白Bcl-2促进细胞内源性凋亡。

MYC蛋白过表达会导致细胞过度增殖、凋亡抑制、代谢重组等促肿瘤效应[22]。最常观察到的导致MYC蛋白过表达的原因为MYC基因重排，在DHL和少数DLBCL病例中，MYC重排是一个决定性特征，此外，MYC基因扩增在2％～20％的DLBCL病例中被观察到[23]–[24]。因此，MYC可作为DLBCL和DHL治疗的潜在靶点。IKZF1和IKZF3属于Ikaros转录因子家族，是参与B细胞分化的转录因子，在针对肿瘤的研究中发现，敲除B细胞淋巴瘤的IKZF1和IKZF3能抑制肿瘤细胞生长，说明B细胞淋巴瘤的发生发展可能与IKZF1/3的失调有关[25]–[26]。因此，通过干预IKZF1、IKZF3或MYC可能具有治疗B细胞淋巴瘤的潜力。我们的研究结果表明，PM能够浓度依赖性地下调DLBCL和DHL细胞系MYC、IKZF1、IKZF3的表达。

综上所述，本研究结果表明PM在体外对DLBCL包括DHL均具有高效的生物活性，具有治疗DLBCL包括DHL的潜力，为PM应用于临床治疗提供了有价值的实验依据。
